# Characterization of In Vivo Keratin 19 Phosphorylation on Tyrosine-391

**DOI:** 10.1371/journal.pone.0013538

**Published:** 2010-10-25

**Authors:** Qin Zhou, Natasha T. Snider, Jian Liao, Daniel H. Li, Anita Hong, Nam-On Ku, Christine A. Cartwright, M. Bishr Omary

**Affiliations:** 1 Department of Medicine, Stanford University School of Medicine, Palo Alto, California, United States of America; 2 Department of Molecular and Integrative Physiology, University of Michigan Medical School, Ann Arbor, Michigan, United States of America; 3 Applied Biomics, Inc., Hayward, California, United States of America; 4 Anaspec, Inc., Fremont, California, United States of America; 5 Department of Biomedical Sciences, Yonsei University, Seoul, South Korea; Cairo University, Egypt

## Abstract

**Background:**

Keratin polypeptide 19 (K19) is a type I intermediate filament protein that is expressed in stratified and simple-type epithelia. Although K19 is known to be phosphorylated on tyrosine residue(s), conclusive site-specific characterization of these residue(s) and identification potential kinases that may be involved has not been reported.

**Methodology/Principal Findings:**

In this study, biochemical, molecular and immunological approaches were undertaken in order to identify and characterize K19 tyrosine phosphorylation. Upon treatment with pervanadate, a tyrosine phosphatase inhibitor, human K19 (hK19) was phosphorylated on tyrosine 391, located in the ‘tail’ domain of the protein. K19 Y391 phosphorylation was confirmed using site-directed mutagenesis and cell transfection coupled with the generation of a K19 phospho (p)-Y391-specific rabbit antibody. The antibody also recognized mouse phospho-K19 (K19 pY394). This tyrosine residue is not phosphorylated under basal conditions, but becomes phosphorylated in the presence of Src kinase in vitro and in cells expressing constitutively-active Src. Pervanadate treatment in vivo resulted in phosphorylation of K19 Y394 and Y391 in colonic epithelial cells of non-transgenic mice and hK19-overexpressing mice, respectively.

**Conclusions/Significance:**

Human K19 tyrosine 391 is phosphorylated, potentially by Src kinase, and is the first well-defined tyrosine phosphorylation site of any keratin protein. The lack of detection of K19 pY391 in the absence of tyrosine phosphatase inhibition suggests that its phosphorylation is highly dynamic.

## Introduction

Intermediate filaments (IFs) encompass a large group of nuclear and tissue-specific cytoplasmic proteins and are major components of the eukaryotic cytoskeleton [1]–[2]. Among the cytoplasmic IFs, keratins (K) are expressed in epithelial cells in a cell-specific manner, and have a characteristic IF molecular structure that consists of a central coiled-coil α helical domain (termed ‘rod’) that is flanked by non-α-helical N-terminal (‘head’) and C-terminal (‘tail’) domains [3]. Keratins include more than 50 unique gene products that are classified into type I (K9-K28, K31-K40) and type II (K1-K8, K71-K86), which associate non-covalently with each other at a 1:1 ratio to form heteropolymers [4]. The various epithelial cell types express specific keratin heteropolymers. For example, keratinocytes preferentially express K5/K14 or K1/K10, depending on their differentiation state in the epidermis, adult hepatocytes express K8/K18 exclusively, and intestinal epithelial cells express K8, along with varying levels of K18/K19/K20 [2]–[3], [5]. K19 is a type I IF protein that is expressed in stratified and simple-type epithelia, such as the small intestine, colon, exocrine pancreas, bladder, gallbladder, and the ductal cells of the liver [6]. It is unique among the other keratins because it has a very short amino acid tail domain [7]–[8].

Appreciation for the physiological significance of keratins is continually growing as a result of the identification of a significant number of human diseases linked to keratin mutations [9]–[13] and the generation of keratin-null and mutant keratin-expressing transgenic mouse lines [14]. Keratins carry out both mechanical and non-mechanical cellular functions, including maintenance of cell integrity, positioning of subcellular organelles, signaling, and protection from injury and apoptosis [3], [15]–[17]. An important mechanism whereby these diverse functions are regulated is via posttranslational modifications, including phosphorylation, which, to date, is the most studied type of modification in keratins [18]–[19]. Keratin phosphorylation is a highly dynamic process that occurs mostly within the ‘head’ and ‘tail’ domains, which harbor most of the structural heterogeneity of these proteins.

Previous work regarding the phosphorylation of K19 demonstrated that head domain residue serine-35 is a major phosphorylation site [20] and that K19 expressed by various cell lines and primary mouse colon epithelial cells undergoes tyrosine phosphorylation upon treatment with pervanadate, a potent tyrosine phosphatase inhibitor [21]. The latter finding is of particular interest since, relative to serine and threonine phosphorylation, tyrosine phosphorylation of IFs is less common, except in the case of vimentin and peripherin [22]–[24], and has not been well characterized in the case of the keratins. In this study we have used molecular, biochemical and immunologic tools to demonstrate that the human K19 tail domain is phosphorylated at tyrosine-391 (Y391) upon phosphatase inhibition in cultured cells and intact tissues. Moreover, Src tyrosine kinase phosphorylates K19 Y391 in transfected cells and in a cell-free system using purified kinase and K19.

## Materials and Methods

### Cells and reagents

HT29 (human colon), NIH-3T3 (mouse fibroblast) and BHK-21 (baby hamster kidney) cells were obtained from the American Type Culture Collection (ATCC). NIH-3T3 cells stably transfected with either vector (pcDNA3.1) or constitutively-active Y527F Src kinase were previously established and described [25]. The antibodies used were: mouse monoclonal antibody (mAb) L2A1, which recognizes human K18 [26], mAb 4.62 (Sigma), which recognizes human K19, and mAb Troma III (University of Iowa Hybridoma Bank) which recognizes mouse K19. The pan anti-phosphotyrosine (pY) antibody used was mAb PY-20 (Invitrogen), and the anti-Src antibody used was mAb-327 (Oncogene). Sodium vanadate (Fisher Scientific, Pittsburg, PA) was used to prepare pervanadate (PV) by mixing with hydrogen peroxide in phosphate-buffered saline (PBS) and used within 1 hr of preparation as described previously [21]. The protein tyrosine phosphatase inhibitors BVT 948, TCS 401, NSC 87877 and NSC 95397 were purchased from Tocris and used as described in the figure legend.

### Mouse experiments

The nontransgenic FVB/n strain (control) and the transgenic mice overexpressing wild type human K19 (hK19; on FVB/n background) were cared for, and used, according to standard institutional guidelines. Pre-sedated three month old mice were injected with 200 µL of saline or PV (5mM vanadate/50 mM H_2_O_2_) rectally by using a flexible plastic tube. The mice were sacrificed one hour later by CO_2_ inhalation and their colons were dissected and divided into 3 parts, which were subsequently used for: (i) Hematoxylin and eosin (H&E) staining, (ii) Immunofluorescence staining, or (iii) Immune blotting. The immunofluorescence and histology staining procedures were carried out as described [27]. All mice received humane care and their use was approved by the Institutional Animal Care Committee at the Palo Alto Veterans Administration (approval number OMA070210MOU).

### Antibody generation and characterization

The rabbit anti-phospho-epitope polyclonal antibody (K19 pY) was generated against the K19 peptide CGQEDH**pY**NNLSA (Anaspec). This peptide corresponds to human pTyr391 and mouse pTyr394-containing K19. The Cys residue, which is not part of the K19 sequence, was added to the N-terminus of the peptide sequence to facilitate coupling to keyhole limpet hemocyanin. Two rabbit antibodies (Ab 3866 and Ab 3867) were generated, but the second and third bleeds of Ab 3866 showed superior reactivity to K19 pY and this antibody was further purified and used for all of the experiments described herein.

### Immunoprecipitation and gel analysis

HT29 cells were solubilized in 1% Nonidet P40 (NP40) in PBS containing 5 mM EDTA and a protease inhibitor cocktail (Sigma). Keratins were immunoprecipitated using antibodies L2A1 and 4.62, and the immunoprecipitates were analyzed by: (i) SDS-polyacrylamide gel electrophoresis (PAGE) followed by transfer to polyvinylidene fluoride (PVDF) membranes for immunoblotting, and by (ii) isoelectric focusing (IEF), SDS-PAGE and transfer to PVDF membranes for immunoblotting.

### Mutagenesis and transfection

Site-directed mutagenesis of human K19 cDNA (GenBank accession number NM_002276.4) to convert Tyr391→Phe, and Tyr4→Phe was performed using QuikChange kit (Stratagene) and confirmed by DNA sequencing. Transfections into BHK-21 and NIH-3T3 cells were performed using lipofectamine reagent (Invitrogen) with wild type (WT) or mutant K19 cDNA plus WT human K8 cDNA (GenBank accession number NM_002273.3). Whole cell lysates from the transfected cells were loaded onto one dimensional (1D) SDS-PAGE or two dimensional (2D) IEF/ SDS-PAGE separation gels for immunoblotting.

### In vitro phosphorylation using Src kinase

Reactions using the Src kinase kit were performed according to the manufacturer's instructions (Millipore). Briefly, immunoprecipitated K19 was added to the kinase buffer containing 8 mM MOPS-NaOH (pH 7.0), 10 mM MnCl2, 1 mM DTT, and 2 µM ATP, in the presence or absence of Src kinase (50 ng) in a total reaction volume of 25 µl. Following an incubation of the reaction mixture at 30°C for 10 min, 2% SDS-containing or urea-containing sample buffer was added for either the 1D or 2D gel electrophoresis analysis, respectively.

## Results

### Identification of Tyr391 as a phosphorylation site in human K19

The NetPhos version 2.0 server (http://www.cbs.dtu.dk/services/NetPhos/) was utilized in predicting putative tyrosine phosphorylation sites in the human K19 protein (K19). The search from this neural network-based method yielded several sites in the head domain (Tyr4, Tyr6 and Tyr61) and one site in the tail domain (Tyr391) of K19 that could potentially undergo phosphorylation (Table S1).

**Figure 1:**
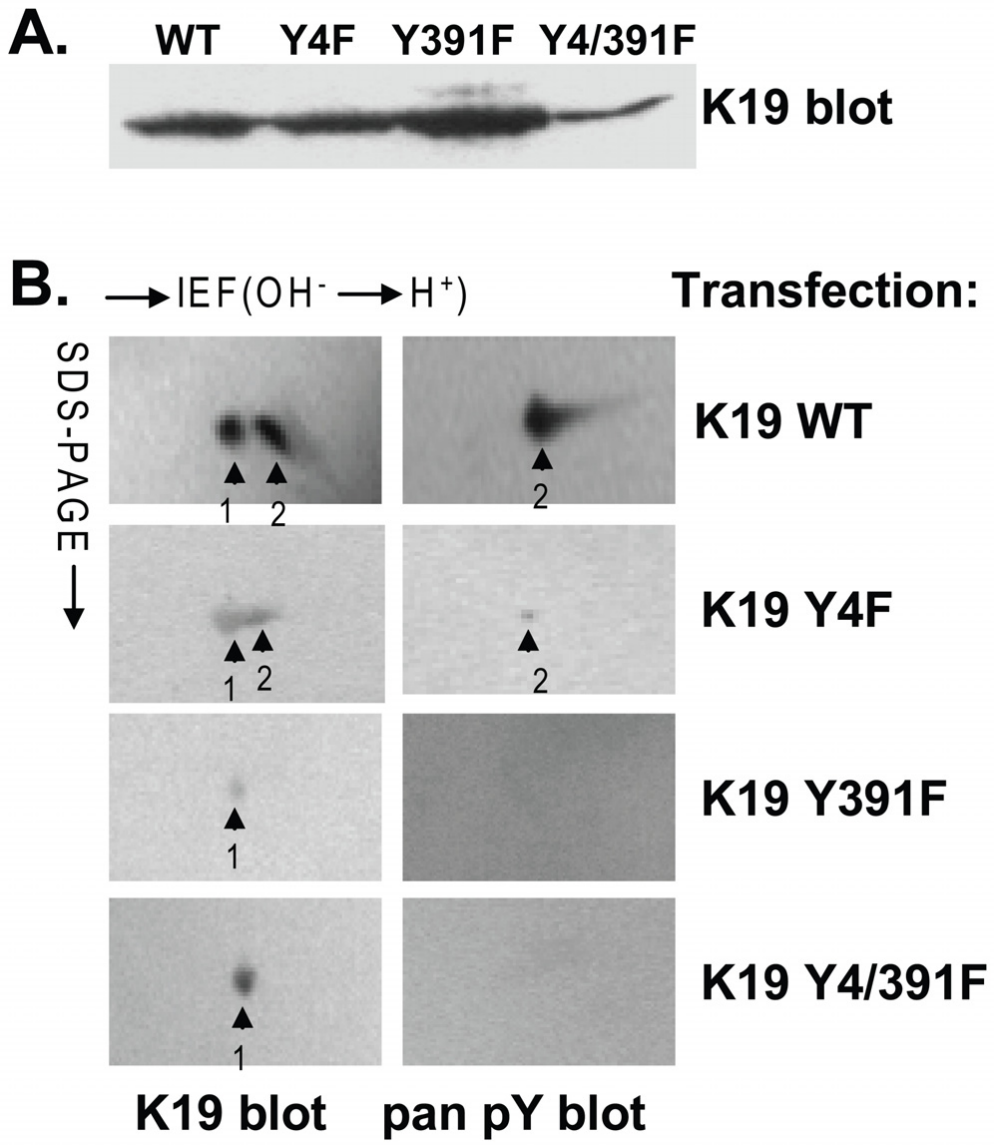
Human K19 is phosphorylated on ‘tail’ domain tyrosine-391. BHK-21 cells were co-transfected with wild type (WT) K8 and one of four K19 constructs (WT, Y4F, Y391F, or Y4,391F). The transfected cells were treated with 1 mM PV for 90 min, then harvested and lysed. **A.** Whole cell lysates were initially analyzed by 1D gel electrophoresis followed immunoblotting using mAb 4.62 (which recognizes total K19) to confirm expression. **B.** For the 2D gel analysis (equal K19 protein loading), isoelectric focusing (IEF) was done in the first dimension followed by SDS page in the second dimension. Immunoblots for total K19 (4.62 mAb) and pan-pY (mAb PY20) are shown and the corresponding phospho-isoforms are labeled as ‘1’ and ‘2’.

Based on the prediction scores that were assigned to the tyrosine residues (Table S1), site-directed mutagenesis was performed on the K19 cDNA to convert Tyr4→Phe (K19 Y4F), Tyr391→Phe (K19 Y391F), and Tyr4,391→Phe (K19 Y4/391F). Following sequencing analysis to confirm the mutation results, BHK-21 cells were transfected with the cDNA of K19 wild type (K19 WT) or one of the mutants. Additionally, the cDNA of wild type human K8 (K8 WT) was co-transfected in each case to allow for stabilization of the transfected keratins as occurs with the normal co-expression of type I and type II keratins [28]. After a 90-min treatment of the transfected cells with pervanadate (PV), an irreversible protein-tyrosine phosphatase inhibitor, whole cell lysates were prepared and subjected to one (1D) or two dimensional (2D) gel electrophoresis and immune blotting using anti-K19 or anti-pan phosphotyrosine (pY) antibodies. Protein expression was confirmed (Figure 1A) and equal amounts of K19 were subjected to 2D analysis.

As demonstrated in Figure 1B, there were two spots detected by the hK19 antibody, including one with an acidic shift (labeled “2”) that was also detected by the anti-pan pY antibody upon analysis of the K19 WT-transfected cell lysates. This was similar to the results obtained with the K19 Y4F-transfected cell lysates (Figure 1), suggesting that Tyr4 in the head domain of K19 is not phosphorylated upon PV cell treatment. In contrast, transfection of either K19 Y391F, or K19 Y4/391F in the BHK-21 cells led to a loss in the phosphotyrosine signal, although a single spot on the 2D gel, representing the presence of K19 protein, was detected in each case. These results demonstrate that Tyr391 in the human K19 protein is phosphorylated in the presence of PV, thereby suggesting that this residue may be phosphorylated under physiological or pathological conditions.

### Characterization of a K19 Tyr-391 using a phospho-epitope-specific antibody

**Figure 2:**
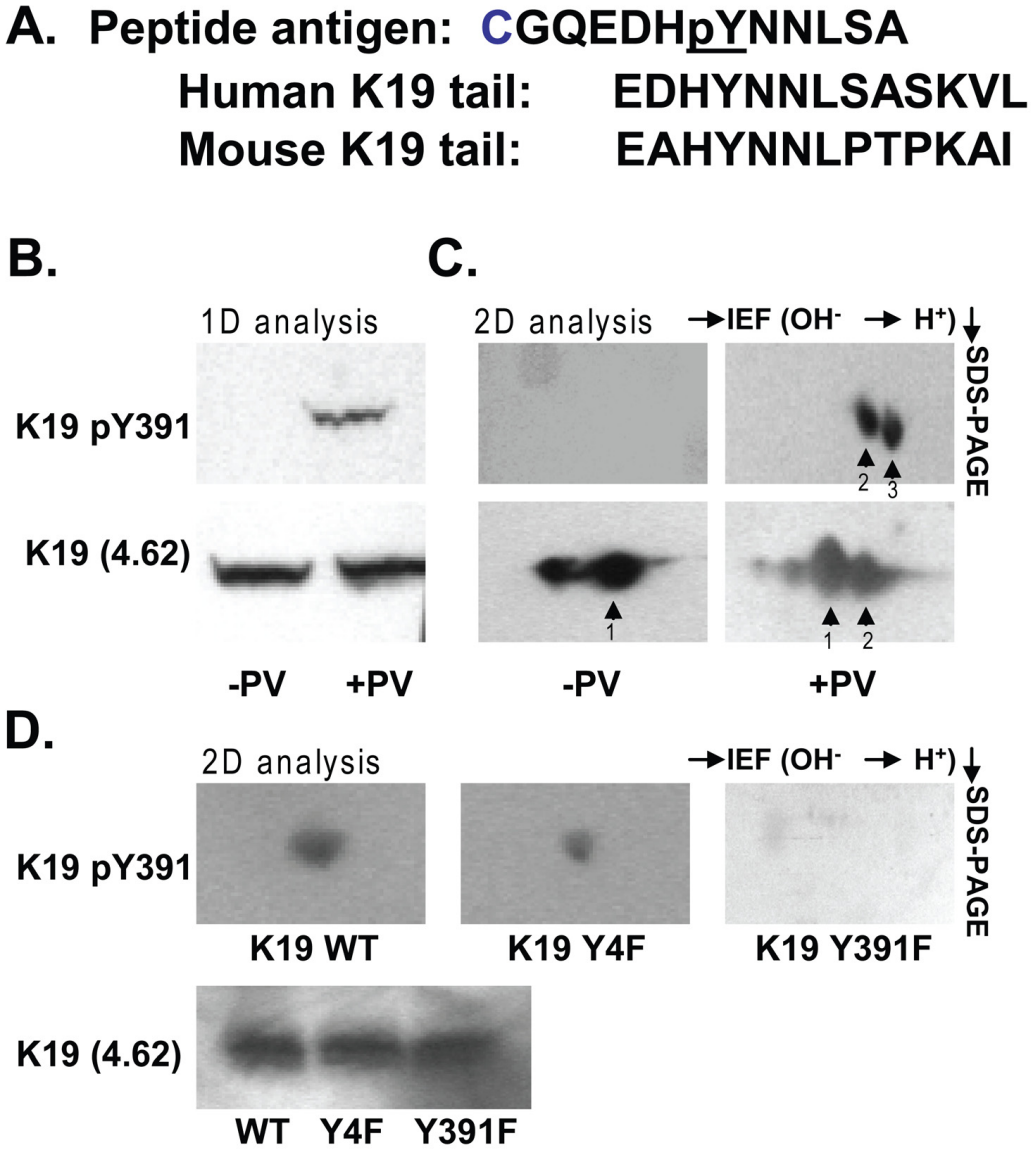
Characterization of a rabbit polyclonal anti-phospho K19 tyrosine 391 (K19 pY391) antibody. **A.** The amino acid sequence of the phospho-peptide used for immunizing rabbits is shown. The peptide antigen is aligned with the human and mouse K19 tail sequences. **B.** Resolution of whole cell lysate proteins from untreated and pervanadate (PV)-treated HT29 cells using standard 1D SDS-PAGE followed by blotting with the antibodies directed towards total K19 (mAb 4.62) and phospho-K19 (rabbit anti-K19 pY391). **C.** The same HT29 cell lysates as those used in panel **B** were resolved using 2D IEF/SDS-PAGE. The K19 pY391 antibody recognizes two acidic K19 isoforms (spots 2 and 3) only in PV-treated, but not control cells. Total K19, as detected by antibody 4.62 is present in control and PV-treated cells. **D.** Whole cell lysates of PV-treated BHK-21 cells transfected with K19 WT, Y4F, or Y391F were resolved using a 1D gel or 2D gel (IEF then SDS-PAGE) and blotted with anti-K19 (to confirm expression; lower panel) and anti-K19 pY391 (upper panels), respectively.

In order to determine more conclusively that Tyr391 is a phosphorylation site in K19, a rabbit antibody specifically recognizing K19 pY391 was developed and characterized. Shown in Figure 2A is the sequence alignment of the human and mouse K19 tail domains with the peptide antigen that was used to generate the anti-K19 pY391 antibody. Due to the very short K19 tail domain, Y391 is in close proximity to the rod domain of the protein.

To test the specificity of the antibody, lysates of either untreated or PV-treated HT29 cells were used in immunoblot experiments with 1D or 2D gel electrophoresis. As shown in Figure 2B, although total K19 (using antibody 4.62) was detected in the lysates from both the untreated and the PV-treated cells, the anti-K19 pY391 antibody specifically recognized a band only in the lysates of the PV-treated cells. Similarly, by using 2D gel electrophoresis to separate the proteins, the anti-K19 pY391 antibody detected a signal in the lysates from the PV-treated cells (dots labeled 2 and 3), but not in the lysates from the untreated cells. As a further confirmation of the specificity of the K19 pY antibody to Tyr391, lysates of PV-treated BHK-21 cells that had been previously transfected with the cDNAs of K8 WT together with, either K19 WT, K19 Y4F, or K19 Y391F, were subjected to western blot analysis. As demonstrated in Figure 2D, the K19 pY signal was absent only in the K19 Y391F- transfected cell lysates, thus confirming the specificity of the K19 pY antibody to Tyr391.

**Figure 3:**
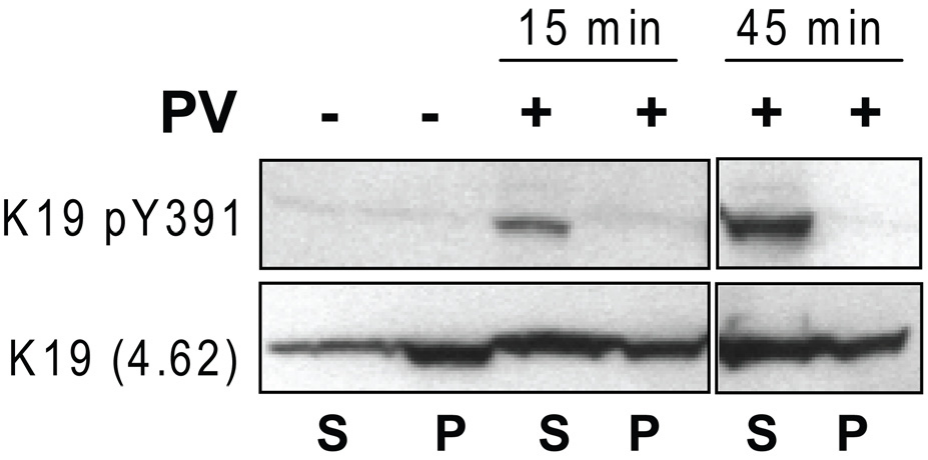
Detergent-based fractionation and distribution of K19 pY391. HT-29 cells were cultured with or without PV treatment for the indicated times. Cells were then solubilized in 1% NP40 for 45 minutes, followed by collection of the supernatant (S) and pellet (P) fractions. Each fraction was suspended in equal volume of sample buffer followed by analysis by SDS-PAGE and immunoblotting using antibodies to the indicated antigens. Note the time-dependent partitioning of K19 pY to the soluble fraction upon treatment with PV.

The anti-K19 pY391 antibody was also used to determine the distribution of K19 pY391 between the soluble and insoluble compartments of HT29 cells, which, respectively, were the supernatant and pellet fractions after lysis of the cells in 1% NP40 detergent [29]. An increase in total K19 is observed in the soluble fraction (antibody 4.62-reactive species) after treatment with PV (Figure 3). K19 pY391 was detected only in the soluble fractions in a time-dependent manner, such that 45 min of PV treatment resulted in significantly more K19 pY391, relative to the shorter treatment of 15 min.

### In vitro phosphorylation of Tyr391 by Src kinase

Since tyrosine phosphorylation of target proteins can be carried out either in a receptor-dependent or receptor-independent manner, a number of potential pathways that could lead to K19 Tyr391 phosphorylation were investigated. Stimulation of several tyrosine kinase receptor pathways in HT29 cells, including the epidermal growth factor receptor and the insulin receptor, did not induce detectable K19 Y391 phosphorylation (data not shown), suggesting a lack of involvement of these two signaling pathways in the context of this cell line.

**Figure 4:**
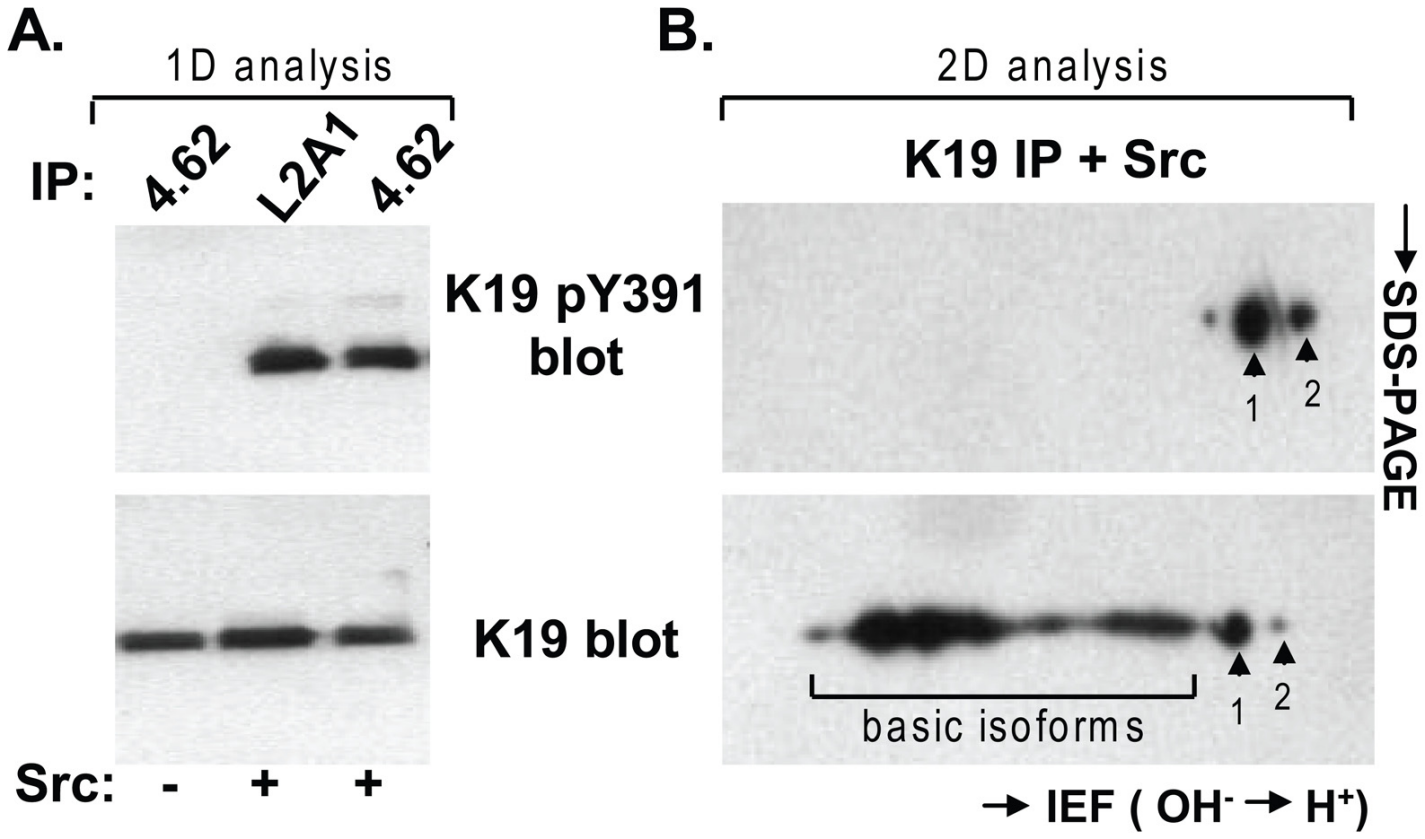
In vitro phosphorylation of K19 by Src kinase. **A.** Keratins were immunoprecipitated from HT29 cells using two different antibodies (4.62 and L2A1). In vitro kinase assays using Src kinase were then carried out as described in Materials and Methods. Samples were analyzed by SDS-PAGE and immunoblotting using antibodies to total K19 (4.62) and to phospho-K19 (anti-K19 pY391). **B.** Kinase reaction products of HT29 K19 immunoprecipitate (using 4.62 antibody) and Src were resolved by 2D IEF/SDS-PAGE gel then blotted for total K19 (4.62 antibody) and K19 pY391. Note that the anti-K19 pY391 antibody does not recognize the more basic K19 isoforms (within bracket) that are recognized by the 4.62 antibody.

In contrast, K19 was phosphorylated on Tyr391 while in the presence of the non-receptor tyrosine kinase Src under both in vitro and in vivo cell culture conditions, as shown in Figure 4. In vitro Src-mediated phosphorylation of K19 was detected upon incubation of human K19, which was immunoprecipitated from HT29 cells using two different antibodies (4.62 and L2A1) (Figure 4A). Additionally, the acidic isoforms reflecting phosphorylated K19 (spots 1 and 2), detected by the anti-K19 pY391 antibody were detected upon 2D gel electrophoresis separation of the in vitro kinase reaction components containing Src and immunoprecipitated human K19 (Figure 4B).

**Figure 5:**
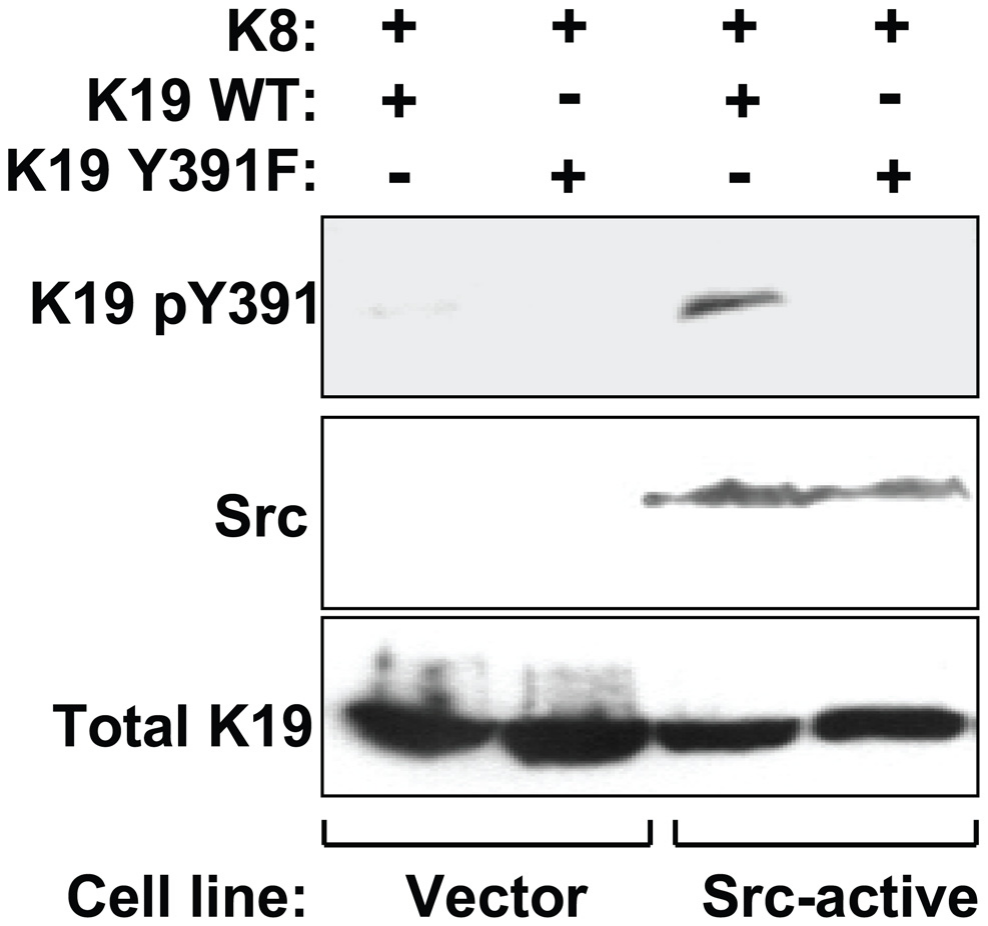
Tyrosine phosphorylation of K19 in NIH-3T3 cells expressing constitutively-active Y527F Src kinase. NIH-3T3 cells stably expressing either empty vector (Vector) or active Src kinase (Src-active) were co-transfected with wild type (WT) K8 and, either K19 WT, or K19 Y391F. Whole cell lysates were prepared from the transfected cells and resolved on SDS-PAGE gels followed by blotting with antibodies to the indicated antigens. Src-dependent phosphorylation of K19 WT, but not K19 Y391F, is observed.

To investigate K19 tyrosine phosphorylation in cell culture conditions, murine fibroblast NIH-3T3 cells stably expressing either constitutively-active Y527F Src, or empty vector, were co-transfected with the DNA of K8 (WT) and K19 (either WT or Y391F). As shown in Figure 5, K19 was phosphorylated at Tyr391 in the Src-expressing cells that also co-expressed the wild type, but not the Y391F, K19 protein. These experiments demonstrate that K19 undergoes Src kinase-mediated phosphorylation at Tyr391 under cell-free and cell culture conditions.

### Exposure of mouse colon to PV increases K19 tyrosine phosphorylation

**Figure 6:**
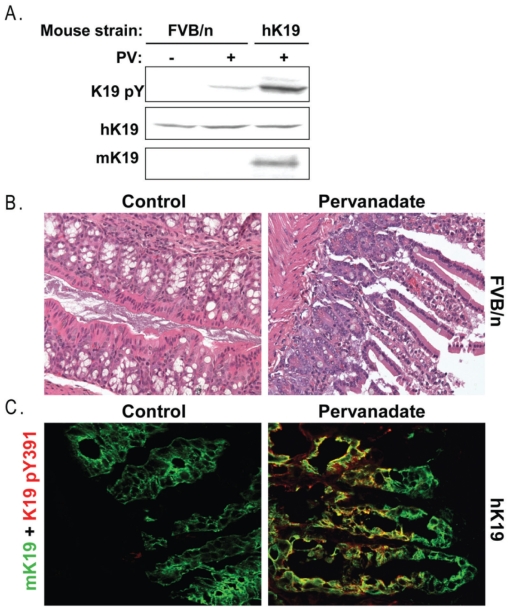
Treatment of mice with pervanadate unmasks tyrosine phosphorylation of K19. Mice were treated with PV as described in Materials and Methods. **A.** Colon tissue lysates (solubilized in 4X SDS-containing sample buffer) from untreated or PV-treated FVB/n (control) and human K19 over-expressing (hK19) transgenic mice were resolved on SDS-PAGE gels and analyzed for the expression of mouse K19 (mK19), human K19 (hK19) and K19 pY391. Note that anti-K19 pY391 also recognizes the corresponding mouse isoform, K19 pY394. **B.** Hematoxylin and eosin staining of colon tissues from untreated or PV-treated FVB/n mice demonstrating PV-induced tissue injury. **C.** The displayed immunofluorescence images represent double-staining of the colons of untreated and PV-treated hK19 mice using antibodies to mouse K19 (mK19) and K19 pY391, which also recognizes the mouse isoform, K19 pY394. Note the yellow staining after PV treatment due to K19 tyrosine phosphorylation after exposure of the colon to PV.

A prior study demonstrated that injection of PV rectally into mice results in significant keratin tyrosine phosphorylation in colon tissue, as determined by using anti-pan pY antibody (22). Therefore, using non-transgenic FVB/n (control) or transgenic mice overexpressing human K19 (hK19), similar experiments were carried out to determine whether Tyr391 of K19 is phosphorylated in tissues after exposure to PV. As shown in Figure 6A, a phosphorylated form of K19 was detected in the colon tissue obtained from both the FVB/n and the hK19 mice treated with PV, but not saline. Hence, the anti-pY391 K19 antibody that was developed also recognizes the corresponding mouse pY394 residue, located in the tail domain (Figure 2A).

As demonstrated by the hematoxylin and eosin (H&E) staining, exposure of mouse colon to PV resulted in edema and injury of the epithelium and breakage of the epithelial cells along the mucosal folds (Figure 6B). Comparison of the immunofluorescence staining of the colon tissue from the hK19 mice treated with either saline (control) or PV, revealed significant up-regulation of K19 pY391/pY394 in epithelial cells that express K19 (Figure 6C). These data demonstrate that mK19 and hK19 undergo pervanadate-dependent phosphorylation in vivo at Tyr394 and Tyr391, respectively.

## Discussion

Intermediate filaments are highly dynamic structures in living cells. The protein subunits are constantly undergoing a process of reversible disassembly and reassembly during the cell cycle and upon a variety of stimuli particularly those related to oxidative stress, apoptosis-inducing or exposure to toxins [30]. One established mechanism by which the keratin assembly and distribution is modulated is via phosphorylation by protein Ser/Thr kinases and dephosphorylation by protein phosphatases to promote, respectively, filament disassembly and reassembly [18]–[19]. In addition to Ser/Thr phosphatase inhibition, Tyr phosphatase inhibition also results in significant keratin filament reorganization although this is related, at least in significant part, to modulation of keratin Ser/Thr phosphorylation [31].

For keratins and other intermediate filament proteins, phosphorylation on tyrosine residues is markedly underrepresented relative to phosphorylation on serine residues. The phosphotyrosine content of epidermal keratins was barely detected when compared with the phosphoserine content [32]. It was previously shown that K8 and K19 are phosphorylated on tyrosine residues after treatment with the potent and irreversible tyrosine phosphatase inhibitor pervanadate, based on metabolic labeling using ^32^PO_4_ and phosphoamino acid analysis, as well as anti-phosphotyrosine antibodies [21]. The findings herein demonstrate that this modification takes place on Tyr391 in the tail domain of human K19 and the corresponding residue, Tyr394, in the tail domain of mouse K19. In addition to mouse and human, this tyrosine residue is conserved in rat (Tyr394), primate (Tyr391), and bovine (Tyr389) K19, but not in chicken (Figure S1), suggesting a possible mammalian-specific role for phosphorylation at this site. Additionally, this modification is likely to be unique to K19 since sequence comparison of human K19 to other type I human keratins shows a lack of conservation for Tyr391. In this study, Tyr391 phosphorylation of human K19 was demonstrated by the use of a phospho-specific antibody recognizing the specific modification in PV-treated cells and mice. Based on in vitro cell-based assays, this phosphorylation could be mediated by Src kinase or possibly a related family member, although the latter was not tested.

Both type I (K18 and K19) and type II (K5-K8) keratins were identified as substrates for oncogenic tyrosine kinases in a large-scale survey of non-small cell lung cancer cell lines and tumors [33]. The mass spectrometry-based phosphoproteomic peptide analysis identified K19 tyrosine-130, located in the rod domain, as the phosphorylation target. Although our initial *in silico* prediction analysis of potential phosphorylation sites identified K19 Y130 as a candidate, it received the second lowest score (Table S1). Therefore, these data would need to be further validated experimentally in order to clarify the physiological significance of K19 pY130.

Despite prior demonstration of tyrosine phosphorylation of few other IFs, the physiological significance of this type of modification still remains to be elucidated. Upon stimulation with platelet-derived growth factor (PDGF), vimentin filaments in porcine aortic endothelial cells undergo reorganization, which is accompanied by an in increase in tyrosine phosphorylation [23]. However, the PDGF-induced filament reorganization was found to be independent of tyrosine phosphorylation. Another potential role for vimentin tyrosine phosphorylation is in chemokine-mediated leukocyte extravasation, as demonstrated in a recent study showing that CCL2 and CCL5 stimulate vimentin tyrosine phosphorylation (the specific tyrosine residues are not known) and filament disassembly, which is partially dependent upon PI3Kγ [24]. However, chemokine-mediated, PI3Kγ -dependent vimentin serine phosphorylation was observed concomitantly with vimentin tyrosine phosphorylation in the context of this study [24], making it difficult to dissect the specific role of each type of modification in this context. Another study has demonstrated that rat PC12 cell peripherin is constitutively phosphorylated at a reported C-terminal tyrosine residue, but this modification is not critical for filament assembly [22] and biochemical confirmation and characterization of phosphorylation at the peripherin terminal tyrosine has not been fully elucidated.

Therefore, IF tyrosine phosphorylation does not appear to play an apparent role in IF network organization, which raises the possibility that this modification serves other functions that remain to be investigated such as the modulation of protein-protein interactions. Such functions are likely to be dynamic since detection of K19 tyrosine phosphorylation under a physiologic or injury-related context has not been possible. For example, tissue damage to the colon and pancreas induced by dextran sulphate sodium (DSS) and a choline-deficient diet (CDD), respectively, as well as exposure of cells to heat, H_2_O_2_, okadaic acid and anisomycin all failed to produce detectable levels of K19 pY391 (data not shown), although such tissue and cell challenges do stimulate marked keratin serine phosphorylation [16], [19]. Additionally, acute treatment of HT29 cells with an alternative general tyrosine phosphatase inhibitor (BVT 948), as well as selective inhibitors of PTP1B (TCS 401), Cdc25 (NSC 95397), and shp1/2 (NSC 87877) protein phosphatases, failed to induce phosphorylation of K19 at tyrosine 391 similar to that observed in the presence of pervanadate (Figure S2). Therefore, the K19 tyrosine phosphorylation described herein does not appear to be regulated by the cellular stress response in the various contexts tested here, although its presence during pervanadate-induced tyrosine phosphatase inhibition suggests that it is highly dynamic.

One plausible hypothesis that remains to be tested is that Src kinase-mediated K19 tyrosine phosphorylation plays a role in the oncogenic effects of this non-receptor tyrosine kinase [34]. The metastatic potential of cancer cells, which correlates with a loss of epithelial differentiation, is augmented in the presence of elevated Src kinase activity [35]. Given that K19 has been shown to be a target of oncogenic kinases in cancer, including Src [33], it is possible that the phosphorylation of K19 Y391 is an event that occurs during epithelial to mesenchymal transition and cancer metastasis. Time-dependence and tissue-specific studies may provide insight into the dynamics of regulation, as well as identify additional tyrosine kinases that may be involved in the phosphorylation of K19. The experimental evidence for Src kinase–mediated phosphorylation of K19 provided herein, along with the availability of the site-specific antibody which recognizes pY391 K19 will enable future testing of this hypothesis.

## Supporting Information

Table S1 Prediction of K19 tyrosine phosphorylation.
(2.12 MB TIF)

Figure S1 Species-specific conservation of K19 Tyr391.
(2.63 MB TIF)

Figure S2 The effect of acute treatment with different protein tyrosine phosphatase inhibitors on K19 Y391 phosphorylation.
(3.01 MB TIF)
