# Supplementary figures and images for: Characterization of In Vivo Keratin 19 Phosphorylation on Tyrosine-391
Source: PLoS One. 2010 Oct 25;5(10):e13538. doi: 10.1371/journal.pone.0013538 (PMC2963603; doi:10.1371/journal.pone.0013538)

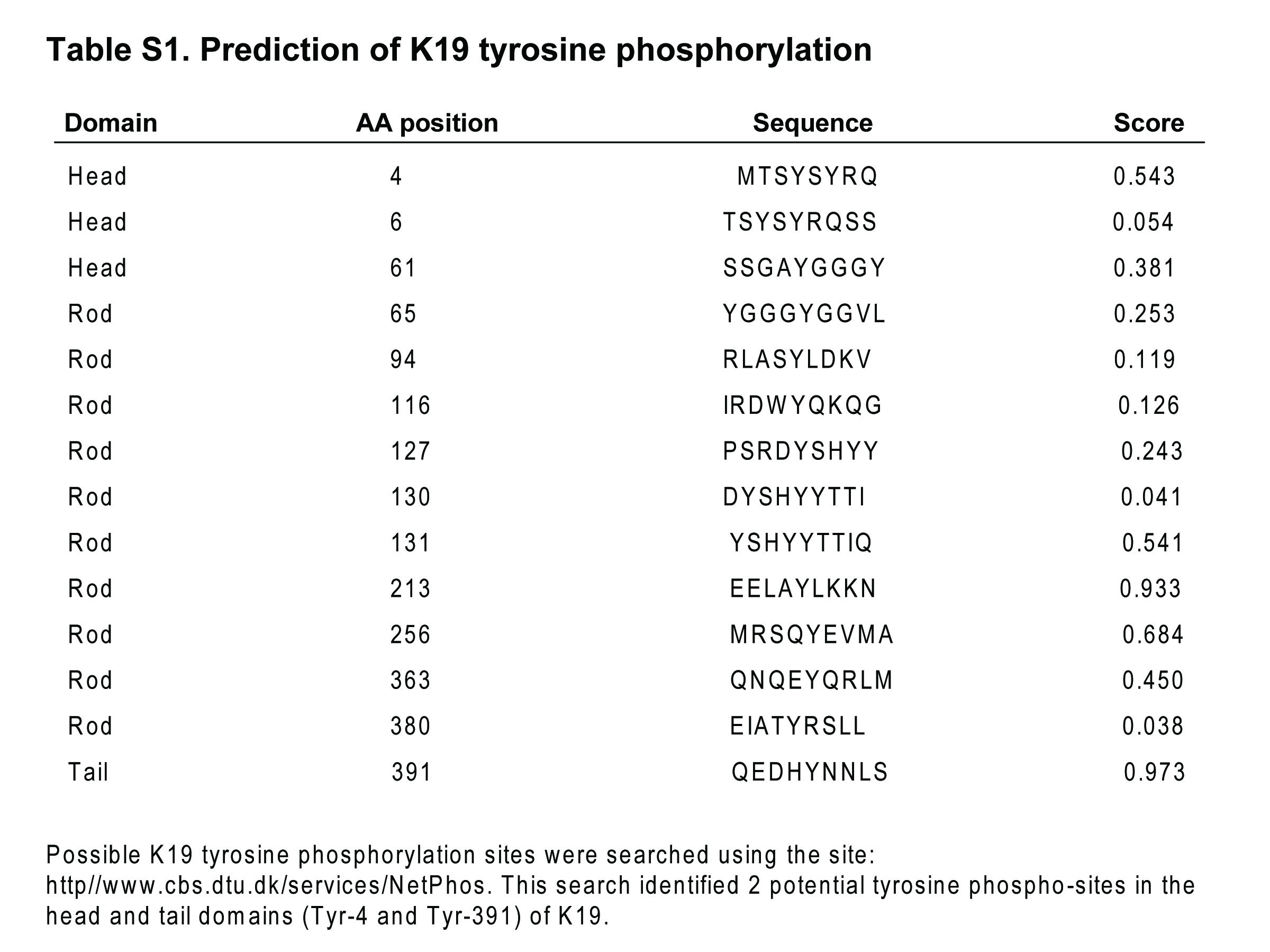

Supplement: Table S1 — Prediction of K19 tyrosine phosphorylation. (2.12 MB TIF) [file pone.0013538.s001.tif]

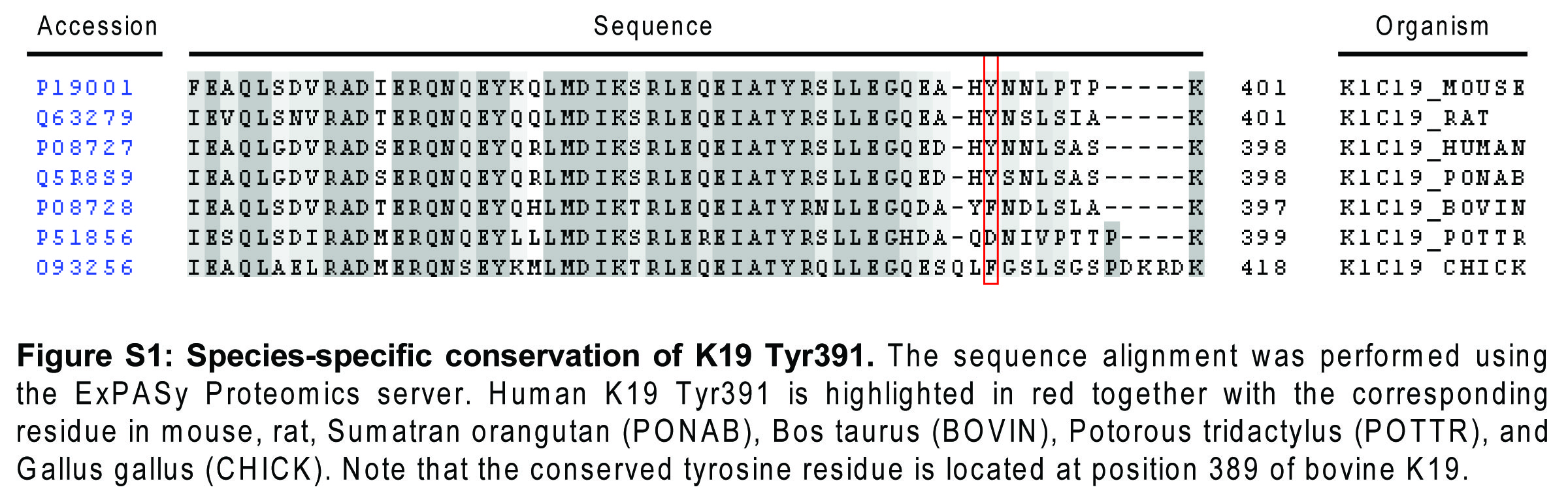

Supplement: Figure S1 — Species-specific conservation of K19 Tyr391. (2.63 MB TIF) [file pone.0013538.s002.tif]

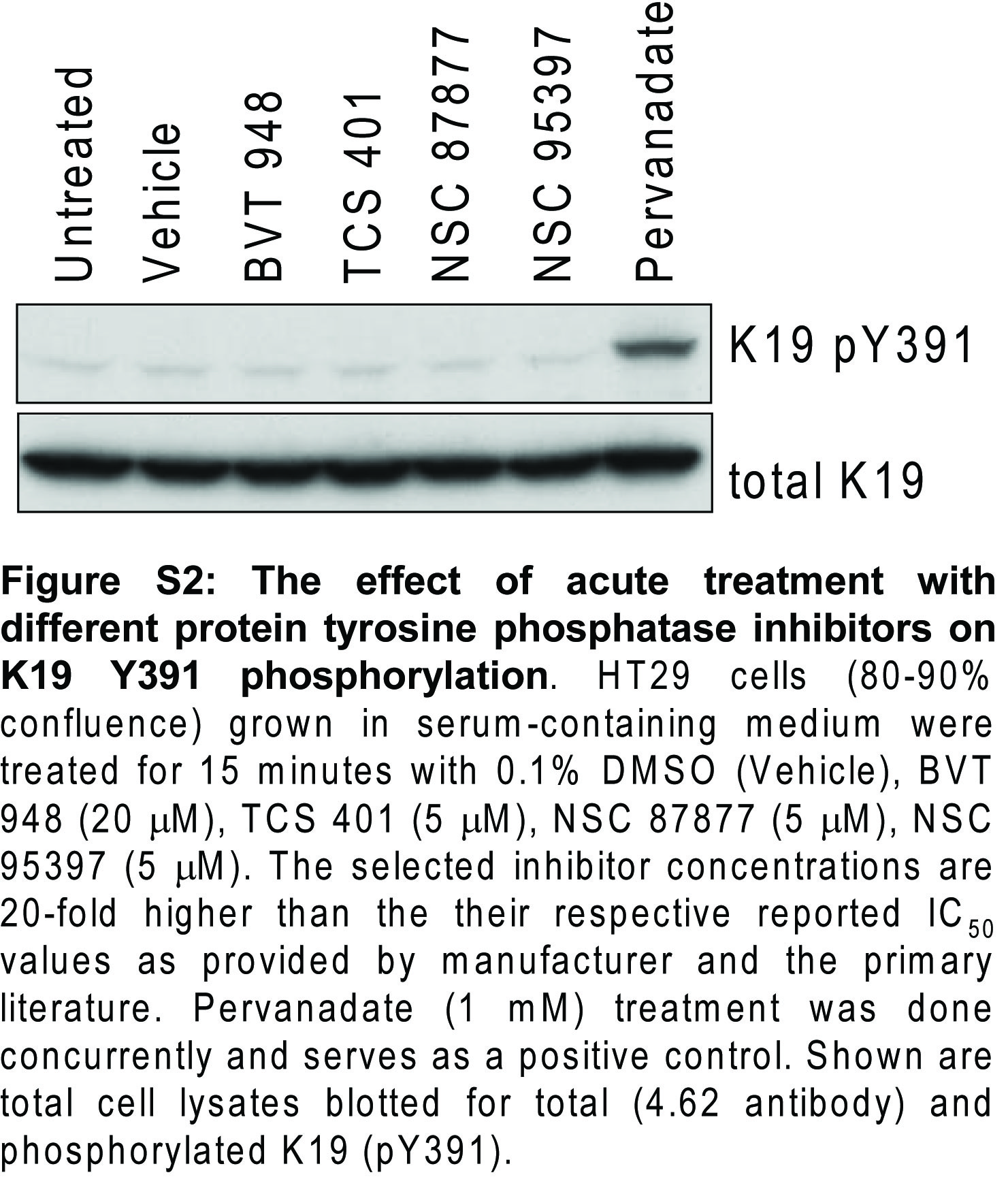

Supplement: Figure S2 — The effect of acute treatment with different protein tyrosine phosphatase inhibitors on K19 Y391 phosphorylation. (3.01 MB TIF) [file pone.0013538.s003.tif]
